# YOSEMITE and RHINE: Phase 3 Randomized Clinical Trials of Faricimab for Diabetic Macular Edema: Study Design and Rationale

**DOI:** 10.1016/j.xops.2021.100111

**Published:** 2021-12-30

**Authors:** Nicole Eter, Rishi P. Singh, Francis Abreu, Kemal Asik, Karen Basu, Caroline Baumal, Andrew Chang, Karl G. Csaky, Zdenka Haskova, Hugh Lin, Carlos Quezada Ruiz, Paisan Ruamviboonsuk, David Silverman, Charles C. Wykoff, Jeffrey R. Willis

**Affiliations:** 1 Department of Ophthalmology, University of Münster, Münster, Germany; 2 Center for Ophthalmic Bioinformatics, Cole Eye Institute, Cleveland Clinic, Cleveland, Ohio; 3 Genentech, Inc., South San Francisco, California; 4 Roche Products (Ireland) Ltd., Dublin, Ireland; 5 New England Eye Center, Department of Ophthalmology, Tufts University School of Medicine, Boston, Massachusetts; 6 Sydney Retina Clinic, Department of Clinical Ophthalmology & Eye Health, University of Sydney, Sydney Eye Hospital, Sydney, Australia; 7 Retina Foundation of the Southwest, Dallas, Texas; 8 Clinica de Ojos Garza Viejo, San Pedro Garza Garcia, Mexico; 9 Department of Medical Policy Development and Strategic Planning, Rajavithi Hospital, College of Medicine, Rangsit University, Bangkok, Thailand; 10 Roche Products Ltd., Welwyn Garden City, United Kingdom; 11 Retina Consultants of Texas, Houston, Texas

**Keywords:** Adjustable dosing, Angiopoietin-2, Anti–vascular endothelial growth factor, Bispecific antibody, Diabetic macular edema, Faricimab, Personalized treatment interval, Phase 3 clinical trial design, AE, adverse event, BCVA, best-corrected visual acuity, CFP, color fundus photography, CRC, central reading center, CST, central subfield thickness, DME, diabetic macular edema, DR, diabetic retinopathy, ETDRS, Early Treatment Diabetic Retinopathy Study, FFA, fundus fluorescein angiography, ITT, intention-to-treat, IxRS, interactive voice or web-based response system, PRN, pro re nata, PTI, personalized treatment interval, SD, spectral-domain, T&E, treat-and-extend, VEGF, vascular endothelial growth factor

## Abstract

**Purpose:**

Faricimab is a novel anti–angiopoietin-2 and anti–vascular endothelial growth factor (VEGF) bispecific antibody with high affinities and specificities for both VEGF and angiopoietin-2. It is postulated that targeting angiogenic factors and inflammatory pathways in addition to the VEGF pathway will increase treatment durability and improve outcomes. The phase 3 YOSEMITE (ClinicalTrials.gov identifier, NCT03622580) and RHINE (ClinicalTrials.gov identifier, NCT03622593) trials are designed to assess efficacy, safety, and durability of faricimab compared with aflibercept in patients with diabetic macular edema (DME). The trials evaluate a personalized treatment interval (PTI) approach to address heterogeneity in treatment response among patients with DME.

**Design:**

Two identically designed, global, double-masked, randomized, controlled phase 3 trials (YOSEMITE and RHINE).

**Participants:**

Adults with center-involving DME secondary to type 1 or 2 diabetes mellitus.

**Methods:**

These studies were designed to evaluate 3 treatment groups: faricimab 6.0 mg dosed either at fixed dosing every 8 weeks after initial treatment with 6 intravitreal doses at 4-week intervals, or faricimab 6.0 mg dosed according to PTI after initial treatment with 4 every-4-week doses, compared with aflibercept 2.0 mg dosed every 8 weeks after 5 initial every-4-week doses. The primary end point of the studies was change from baseline in best-corrected visual acuity at 1 year, averaged over weeks 48, 52, and 56. Secondary end points included anatomic, durability, and patient-reported outcomes. Safety outcomes included incidence and severity of ocular and nonocular adverse events. The PTI is a protocol-defined flexible regimen based on the treat-and-extend concept, which allowed up to every-16-week adjustable dosing based on objective and standardized criteria. The PTI design aimed to maximize therapeutic results while minimizing treatment burden.

**Main Outcome Measures:**

We describe the rationale for the study design and the novel PTI (up to every-16-week adjustable dosing) approach for treatment with faricimab.

**Results:**

YOSEMITE and RHINE enrolled 940 and 951 patients, respectively. Results from each study will be reported separately.

**Conclusions:**

YOSEMITE and RHINE were the first registrational trials in retinal disease to incorporate an objective PTI regimen, allowing for up to every-16-week adjustable dosing with a dual angiopoietin-2 and VEGF-A inhibitor, faricimab 6.0 mg, for treatment of DME.

Intravitreally administered anti–vascular endothelial growth factor (VEGF) agents have demonstrated efficacy in treating diabetic macular edema (DME) and improving visual acuity in phase 3 trials.^1^, ^2^, ^3^, ^4^, ^5^, ^6^ However, data generated outside clinical trials suggest that the frequent clinical evaluations and associated vision gains reported in clinical trial settings are difficult to achieve, to maintain, or both in routine clinical practice.^7^^,^^8^ Personalized treatment regimens, such as treat-and-extend (T&E) and pro re nata (PRN; i.e., treat as needed), are often used to reduce treatment burden associated with fixed-interval (every 4 or 8 weeks) intravitreal injections, and these approaches may also address the heterogeneity in individual anti-VEGF response.^9^, ^10^, ^11^ Although PRN regimens reduce the need for injections, frequent office visits are still required, whereas T&E approaches can extend monitoring intervals and reduce the number of office visits.^12^ However, a major knowledge gap is the efficacy of a personalized treatment approach for DME as evaluated in a double-masked, global, registrational trial. YOSEMITE (ClinicalTrials.gov identifier, NCT03622580) and RHINE (ClinicalTrials.gov identifier, NCT03622593) were the first global, double-masked, randomized trials to objectively evaluate a personalized treatment interval (PTI) regimen for DME based on the T&E concept. These phase 3 trials evaluated the efficacy, safety, and durability of treatment with faricimab, an angiopoietin-2 and VEGF-A dual-pathway inhibitor, compared with the anti-VEGF agent, aflibercept. Herein, we describe the design of the YOSEMITE and RHINE trials and explain the rationale for the unique features of these studies, which included a PTI (with up to every-16-week adjustable dosing) approach for treatment with faricimab.

## YOSEMITE and RHINE Study Design and Rationale

### Study Overview

The YOSEMITE and RHINE trials are 2 identically designed, double-masked, multicenter, randomized, parallel-group, registrational phase 3 studies of faricimab in patients with DME. The studies were designed to evaluate the efficacy, safety, pharmacokinetics, and durability of intravitreal faricimab 6.0 mg for the treatment of DME when dosed either every 8 weeks or according to a PTI regimen in adjustable intervals (up to every 16 weeks), compared with intravitreal aflibercept 2.0 mg dosed every 8 weeks as per the label. Input from global health authorities on the design of the studies was obtained, and the data generated were used to support a potential marketing authorization application for faricimab in DME. These studies were conducted in accordance with the International Conference on Harmonisation E6 Guideline for Good Clinical Practice and the principles of the Declaration of Helsinki, or the laws and regulations of the country in which the research was conducted. Written informed consent was obtained before initiation of any study procedures, and the study protocol was approved by institutional review boards before study start (Supplementary Table 1 includes the information for the 179 institutional review boards).

**Figure 1:**
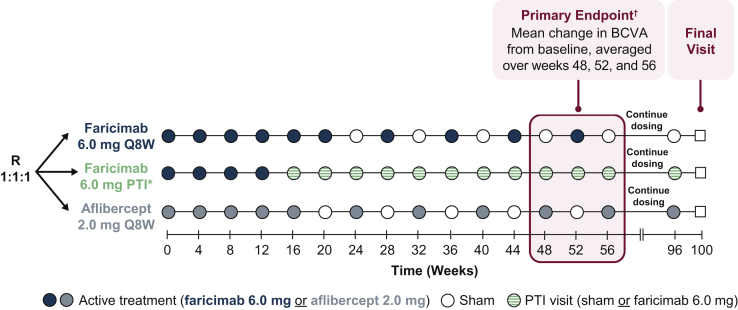
Diagram showing study design overview. ∗The personalized treatment interval (PTI) is a protocol-driven regimen based on the treat-and-extend concept. ^†^Change from baseline in best-corrected visual acuity (BCVA), as measured on the ETDRS chart at a starting distance of 4 m at 1 year, is the average of the week 48, 52, and 56 visits. Q8W = every 8 weeks; R = randomization.

The YOSEMITE and RHINE trials enrolled 1891 patients across 31 countries (YOSEMITE, 940 patients across 179 centers; RHINE, 951 patients across 174 centers). The studies comprised 3 treatment arms: (1) faricimab 6.0 mg monthly (every 4 weeks) for 6 months followed by every-8-week dosing; (2) faricimab 6.0 mg every 4 weeks for 4 months followed by per PTI, a protocol-driven T&E regimen with up to every-16-week dosing; or (3) aflibercept 2.0 mg every 4 weeks for 5 months followed by every-8-week dosing, in line with the product label^13^ (Fig 1). Patients were randomized 1:1:1 to each of the 3 treatment arms of the studies (Fig 1). Randomization was stratified by baseline best-corrected visual acuity (BCVA) Early Treatment Diabetic Retinopathy Study (ETDRS) letter score (64 ETDRS letters or better vs. 63 letters or worse; Snellen equivalent threshold, ∼20/63), prior intravitreal anti-VEGF therapy (yes vs. no), and region (United States and Canada, Asia, and the rest of the world). The goal of stratification was to prevent imbalance of these potentially confounding variables across the study arms that could affect the interpretation of study outcomes.

The primary end point analysis of the studies was conducted at 1 year, and the total duration of the core studies was 2 years. To preserve masking, patients were seen every 4 weeks and underwent a sham procedure at study treatment visits when they were not treated with active study drug.

### Study Participants and Eligibility Criteria

General inclusion and exclusion criteria are shown in full in Supplementary Table 2. In brief, patients 18 years of age or older with center-involving DME secondary to type 1 or type 2 diabetes mellitus were eligible to participate. The inclusion criterion for hemoglobin A1c level was set at up to 10% to limit enrollment of patients with unstable diabetic control to minimize any potential changes to the outcome variables that could be secondary to large fluctuations in underlying glucose levels. General exclusion criteria included, among others, untreated diabetes or treatment initiated within 3 months of day 1; uncontrolled high blood pressure; and history of other disease, physical examination finding, or clinical laboratory finding suggestive of a condition that would contraindicate use of any of the study drugs, may affect interpretation of the study results, or in the opinion of the investigator would render the patient at high risk for treatment complications.

One eye per patient was designated as the study eye. Ocular exclusion and inclusion criteria for the study eye are shown in Table 1. The central reading centers (CRCs) evaluated the spectral-domain (SD) OCT and color fundus photography (CFP) images obtained at screening to provide an objective, masked assessment of whether patients’ study eyes met the study eligibility criteria. If both eyes were eligible for inclusion, the eye with the worse BCVA at screening was selected as the study eye.

**Table 1:** Ocular Exclusion and Inclusion Criteria for the Study Eye

Exclusion Criteria	Inclusion Criteria
• High-risk PDR in the study eye (any vitreous or preretinal hemorrhage; neovascularization elsewhere one-half disc area or more within an area equivalent to the mydriatic ETDRS 7 fields on clinical examination or on CFP images; neovascularization at disc one-third disc area or more on clinical examination), as graded by the CRCs • Tractional retinal detachment, preretinal fibrosis, or epiretinal membrane involving the fovea or disrupting the macular architecture in the study eye • Active rubeosis • Uncontrolled glaucoma • History of retinal detachment or macular hole (stage 3 or 4) • Aphakia or implantation of anterior chamber intraocular lens • Intravitreal anti-VEGF treatment within 3 months∗ (previously treated patients) or any intravitreal anti-VEGF agents in study eye before day 1 (treatment-naïve patients) • Treatment with PRP within 3 mos∗ • Macular (focal or grid) laser within 3 mos∗ • Any cataract surgery or treatment for complications of cataract surgery with steroids or YAG laser capsulotomy within 3 mos∗ • Any other intraocular surgery • Any intravitreal or periocular (sub-Tenon) corticosteroid treatment within 6 mos∗ • Any use of medicated intraocular implants, including Ozurdex (Allergan USA, Inc., Madison, NJ), within 6 mos∗ • Any use of Iluvien implants at any time • Treatment for other retinal diseases that can lead to macular edema	• Macular thickening secondary to DME involving the center of the fovea, with CST ≥325 μm (defined as the thickness from the ILM to Bruch’s membrane), measured by SD OCT or SS OCT (Spectralis [Heidelberg Engineering GmbH, Heidelberg, Germany], Topcon [Topcon, Tokyo, Japan], or Cirrus [Carl Zeiss Meditec, Dublin, CA]) in the central 1-mm area of the macula as graded by the CRCs • BCVA between 25 and 73 ETDRS letters (approximate Snellen equivalent, 20/320–20/40), as assessed on the standardized ETDRS chart at 4 mos • Sufficiently clear ocular media and adequate pupillary dilatation to allow acquisition of good-quality CFP images (including ETDRS 7 modified fields or 4 wide-angle fields to permit grading of DR and assessment of the retina) and other imaging methods

BCVA = best-corrected visual acuity; CFP = color fundus photography; CRC = central reading center; CST = central subfield thickness; DME = diabetic macular edema; DR = diabetic retinopathy; ILM = internal limiting membrane; PDR = proliferative diabetic retinopathy; PRP = panretinal photocoagulation; SD = spectral-domain; SS = swept-source; VEGF = vascular endothelial growth factor; YAG = yttrium–aluminum–garnet.

∗ Before day 1 of study.

Study eyes were permitted to be either anti-VEGF treatment naïve (with no previous history of intravitreal anti-VEGF therapy) or previously anti-VEGF treated (provided that the last treatment was ≥3 months before the day 1 study visit). Study eyes previously treated with anti-VEGF therapy were capped at 25% of the total patient enrollment for each study. The rationale for capping the number of patients previously treated with anti-VEGF therapy was based on the heterogeneous nature of this patient population, with a potential history of long-standing and potentially insufficiently treated DME, resulting in pharmacologically irreversible macular damage that could thus limit the possibility of visual acuity improvements.

### Anatomic Assessments

Retinal anatomic features were evaluated with OCT (SD OCT or swept-source OCT), CFP, fundus fluorescein angiography (FFA), and optional OCT angiography images. Because of the global nature of the trials, the study used 2 CRCs (Duke Reading Center and Vienna Reading Center) to collect all OCT, CFP, FFA, and optional OCT angiography images. The Duke and Vienna CRCs graded all OCT images, as well as the optional OCT angiography images. The CFP and FFA images were transferred to the third reading center (Wisconsin Fundus Photograph Reading Center), which was not in direct contact with the study sites and served as the evaluator of all CFP and FFA images. To ensure that OCT assessments were reproducible and replicable by both the Duke and Vienna CRCs, a harmonization process between these 2 reading centers was undertaken that yielded excellent grading concordance in image assessments overall; an article on this exercise will be published elsewhere.

### Rationale for Choice of Comparator and Comparator Dosing

The YOSEMITE and RHINE trials were designed as active comparator–controlled studies. The efficacy of 2 regimens of faricimab 6.0 mg was compared with the intravitreal anti-VEGF agent, aflibercept 2.0 mg. Eyes in the aflibercept arm (Fig 1) were treated with 5 every-4-week initial doses, followed by maintenance doses of aflibercept 2.0 mg every 8 weeks to week 96, with assessments up to the final study visit at week 100. Given the registrational potential of the trials, a comparator was needed with a globally aligned posology that would be acceptable to health authorities around the world. The aflibercept dosing regimen used in the trials is aligned with globally approved posology, which, unlike ranibizumab, has different doses and injection regimen approvals across geographical regions; the same dose of aflibercept 2.0 mg and the same every-8-week maintenance regimen have been approved globally for the treatment of patients with DME, facilitating the use of aflibercept as a comparator in the global trial setting.^13^, ^14^, ^15^ The decision to use aflibercept as a comparator aligned well with the findings from the 2020 American Society of Retina Specialists Preferences and Trends survey, which reported that aflibercept was the agent that retina specialists most commonly use as first-line therapy.^16^

### Rationale for the Faricimab Fixed-Interval Dosing Arm

Patients in the faricimab fixed-interval every-8-week regimen (Fig 1) received initial dosing with 6 intravitreal injections of faricimab 6.0 mg at every-4-week intervals, based on the treatment regimen and efficacy results of the phase 2 BOULEVARD trial,^17^ in which patients treated with faricimab showed continuous BCVA improvement with each every-4-week dose up to week 24.

The use of an every-8-week maintenance interval in this arm was supported by durability evidence from the off-treatment observation period in the BOULEVARD trial, which demonstrated that 93% of patients showed no disease reactivation 8 weeks after the last dose of faricimab 6.0 mg.^17^ Furthermore, pharmacokinetic and pharmacodynamic assessments of aqueous humor samples from a subset of patients treated with faricimab in the AVENUE phase 2 trial^18^ showed suppression of ocular-free angiopoietin-2 and VEGF-A for at least 8 weeks.^19^

### Objective and Rationale for the Faricimab Personalized Treatment Interval Arm

The objective of the PTI approach, based on the T&E concept, was to achieve maximum vision gains while minimizing the burden of frequent visits and treatment by tailoring the frequency of intravitreal injections to the individual’s anatomic, functional, and visual responses to treatment. It was developed by taking into account key learnings from previous studies that evaluated T&E approaches in treating patients with DME.

The first randomized, global, multicenter, controlled trial that compared T&E and PRN regimens with ranibizumab 0.5 mg in patients with DME was the RETAIN phase 3 trial.^12^ This study demonstrated noninferiority of BCVA change from baseline with T&E versus PRN ranibizumab at year 2. Both regimens involved treatment decisions based on anatomic (via OCT) and BCVA criteria. The results showed that the number of required office visits with the T&E regimen was 46% lower than with the PRN regimen in patients with DME over a 2-year period, with approximately 70% of patients in the T&E group achieving monitoring intervals of 2 months or more.^12^ A further randomized controlled trial to assess a T&E strategy for ranibizumab 0.3 mg in DME was the TREX-DME trial.^11^ This study tested a unique T&E concept, using central retinal thickness changes as the primary driver of dosing interval determination. The TREX-DME trial showed that an effective T&E dosing regimen could be based on an algorithm largely driven by central retinal thickness measurements to provide similar visual outcomes as monthly dosing.^11^ Both these T&E studies in DME demonstrated that the T&E approach may be both feasible to reproduce in the real world and may be an effective way to reduce the need for frequent office visit assessments, while achieving optimal outcomes. However, limitations of these T&E approaches included the lack of double masking and introducing subjectivity into the determination of disease activity by the assessing clinician. In the TREX-DME trial, the assessment of OCT values relied on evaluation at the study site level and did not use a CRC connected to an interactive voice or web-based response system (IxRS) system that could calculate OCT changes automatically over time in a standardized manner.

Therefore, for the phase 3 studies of faricimab in DME, to eliminate bias and to ensure robust and reproducible results, we aimed to develop a PTI regimen based on T&E methodology. This PTI regimen used an automated treatment algorithm that generated individualized treatment schedules for every patient based on standardized, objectively measured clinical parameters.

The faricimab PTI regimen (Fig 1) was protocol determined, automated, standardized, and objective and was based on the T&E concept, with dosing intervals adjusted by 4-week intervals according to prespecified BCVA and central subfield thickness (CST) criteria, defined as the central 1-mm thickness from the internal limiting membrane to Bruch’s membrane, as measured on SD OCT or swept-source OCT. Described in more detail below, the PTI allowed for up to every-16-week adjustable dosing intervals.

The efficacy outcomes from the BOULEVARD trial suggested heterogeneity in treatment responses to dual inhibition of angiopoietin-2 and VEGF-A with faricimab among patients with DME, as is also observed with intravitreal injections of anti-VEGF treatment.^20^ However, time to disease reactivation data from the off-treatment observation period of the BOULEVARD trial showed that although a small proportion of patients may require every-4-week dosing, faricimab could potentially enable most patients to be dosed on an every-12-week or every-16-week maintenance dosing regimen.^17^ Therefore, the PTI arm (Fig 1) was designed so that individuals could receive treatment as frequently as every 4 weeks or up to every 16 weeks, depending on their clinical (BCVA and OCT findings) response to treatment with faricimab. Intervals beyond every 16 weeks were not studied in the BOULEVARD trial.^17^

To allow for objective, unbiased assessment, masked graders at the Duke and Vienna CRCs received the OCT images from the sites and evaluated CST for all patients in all arms at every study visit and entered these values into the IxRS system. The ETDRS BCVA values were entered directly into the IxRS system by site staff. Treatment interval decisions in the PTI arm were then calculated automatically by the IxRS algorithm. Specifically, IxRS used BCVA and CST data from active dosing visits and not those from sham visits to determine whether a patient’s existing treatment interval should be reduced by 4 or 8 weeks, maintained, or extended by 4 weeks, up to a maximum of 16 weeks. This was to replicate what would happen in a setting outside clinical trials, where only data from treatment visits would be used.

### Personalized Treatment Interval Algorithm

Patients randomized to the PTI arm (Fig 1) received 4 initial monthly doses of faricimab 6.0 mg at fixed every-4-week intervals (until at least the week 12 visit). At or after week 12, as soon as the patient reached CST of < 325 μm, the dosing interval could be adjusted gradually upward by 4-week increments, as described below, to a maximum interval of every 16 weeks, with the option to drop back by 4 or 8 weeks according to individual patient needs. The selection of 4 initial doses aimed to balance visual acuity outcomes while giving patients the opportunity to receive fewer intravitreal injections during the first phase of treatment. This CST threshold was chosen because it reflects how the DME study population was defined at screening in both this study and in the TREX-DME trial.^11^ Thereafter, the treatment interval was either extended in 4-week intervals, was reduced in 4- or 8-week intervals, or was maintained based on changes in CST and BCVA values obtained at subsequent active study drug visits (i.e., not at sham treatment visits). The algorithm is described in more detail below. The maximum dosing interval was every 16 weeks and the minimum was every 4 weeks. Intervals beyond every 16 weeks were not studied.

**Figure 2:**
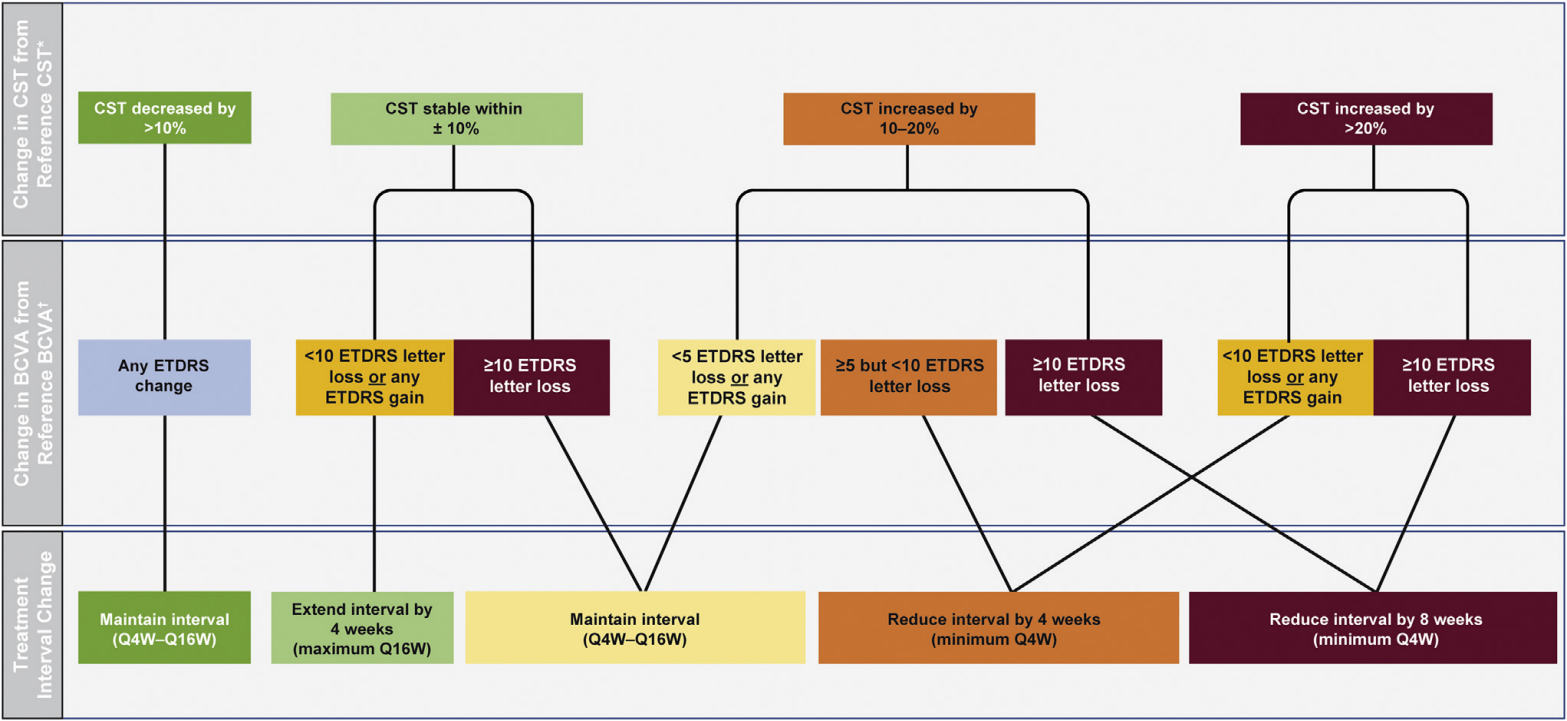
Decision tree for interactive voice or web-based response system–determined personalized treatment interval arm dosing intervals. ∗The first central subfield thickness (CST) value that is < 325 μm (defined as the central 1-mm thickness from the internal limiting membrane to Bruch’s membrane), starting at week 12. Reference CST is adjusted if CST decreases by more than 10% from the previous reference CST for 2 consecutive study drug dosing visits and the values obtained are within 30 μm. The CST value obtained at the latter visit serves as the new reference CST. ^†^The mean of the 3 best best-corrected visual acuity (BCVA) scores obtained at any previous active dosing visit. Q4W = every 4 weeks; Q16W = every 16 weeks.

The PTI algorithm was fully automated and was based primarily on changes in CST measurements, with adjustments determined by the direction and degree of BCVA change (Fig 2; Table 2). The changes in CST and BCVA were calculated relative to their reference values. The reference CST was defined as the first CST measurement less than the 325-μm threshold (at week 12 or later, at active drug study visits). During the PTI phase, reference CST was adjusted if CST decreased by more than 10% from the previous reference CST for 2 consecutive study drug dosing visits and the values obtained were within 30 μm of each other. The CST value obtained at the later of 2 such visits served as the new reference CST. The reference BCVA was defined as the average of the 3 best BCVA values obtained at prior active study drug visits.

**Table 2:** Algorithm for Interactive Voice or Web-Based Response System–Determined Personalized Treatment Interval Arm Dosing Intervals, Initiated after Diabetic Macular Edema Was Clinically Controlled (<325 μm)

Treat-and-Extend Principle	Change in Treatment Intervals, as Determined by Change in Reference Central Subfield Thickness∗ and Best-Corrected Visual Acuity† Values			
	Interval Extended by 4 Wks	Interval Maintained	Interval Reduced by 4 Wks	Interval Reduced by 8 Wks
Rationale for decision	As soon as DME is stable,‡ increase treatment interval when anatomic features are relatively unchanged	As soon as DME is stable,‡ maintain existing interval if interval extension and reduction criteria are not met	Reduce existing treatment interval when evidence exists that it is associated with worsening of anatomic features, vision, or both	Significantly reduce existing treatment interval when evidence exists that it is associated with worsening of anatomic features and significant vision loss
Criteria	CST value is increased or decreased by ≤10% without an associated ≥10-letter BCVA decrease	CST value is increased or decreased by ≤10% with an associated ≥10-letter BCVA decrease or	CST value is increased between >10% and ≤20% with an associated ≥5- to <10-letter BCVA decrease or	CST value is increased by >20% with an associated ≥10-letter BCVA decrease
		CST value is increased between >10% and ≤20% without an associated ≥5-letter BCVA decrease CST value is decreased by >10%§	CST value is increased by >20% without an associated ≥10-letter BCVA decrease	

BCVA = best-corrected visual acuity; CST = central subfield thickness; DME = diabetic macular edema.

∗ The first CST value that is < 325 μm (defined as the central 1-mm thickness from the internal limiting membrane to Bruch’s membrane), starting at week 12. Reference CST is adjusted if CST decreases by more than 10% from the previous reference CST for 2 consecutive study drug dosing visits and the values obtained are within 30 μm. The CST value obtained at the latter visit serves as the new reference CST.

† The mean of the 3 best BCVA scores obtained at any previous study drug dosing visit.

‡ The underlying macular edema was deemed clinically controlled when CST values of < 325 μm were achieved. This threshold reflects how the DME study population was defined at screening in both this study and in the TREX-DME trial.

§ Anatomic improvements suggest ongoing benefit.

**Figure 3:**
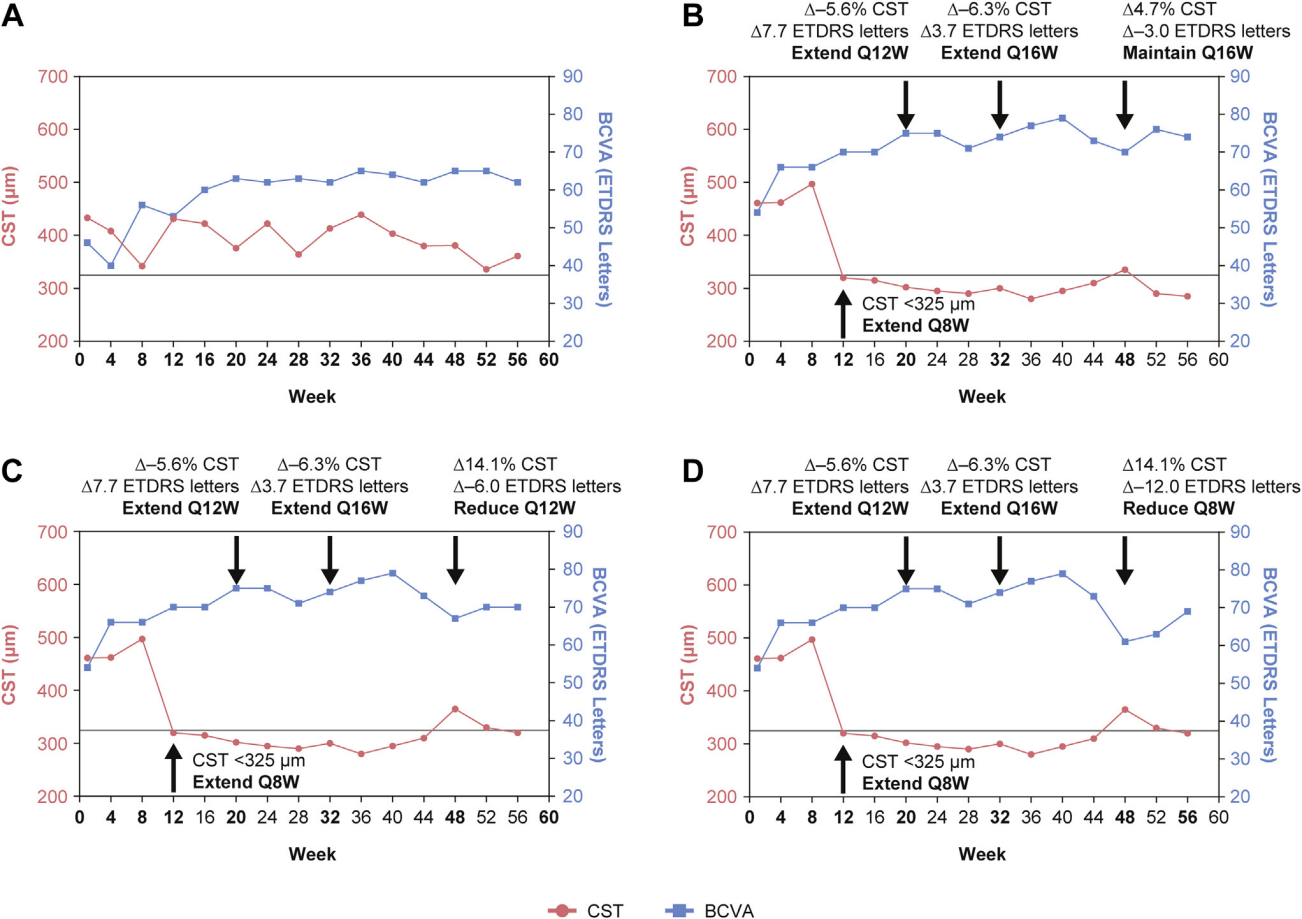
Graphs showing personalized treatment interval scenario examples. Horizontal line represents central subfield thickness (CST) threshold of 325 μm. Weeks in boldface indicate when active treatment was administered. **A**, Central subfield thickness threshold of < 325 μm (represented by solid horizontal line) not met: patient continues every-4-week (Q4W) treatment. **B**, Week 12: CST < 325 μm, extend to every 8 weeks (Q8W); week 20: CST within ±10% of reference CST∗ (with no associated ≥10-letter best-corrected visual acuity [BCVA] decrease from reference BCVA^†^), extend to every 12 weeks (Q12W); week 32: within ±10% of reference CST (with no associated ≥10-letter BCVA decrease from reference BCVA), extend to every 16 weeks (Q16W); week 48: CST within ±10% of reference CST (with no associated ≥10-letter BCVA decrease from reference BCVA), maintain at Q16W. **C**, Week 12: CST < 325 μm, extend to Q8W; week 20: CST within ±10% of reference CST (with no associated ≥10-letter BCVA decrease from reference BCVA), extend to Q12W; week 32: CST within ±10% of reference CST (with no associated ≥10-letter BCVA decrease from reference BCVA), extend to Q16W; week 48: CST increased by more than 10%, but no more than 20% (with an associated ≥5-letter to <10-letter BCVA decrease), reduce to Q12W. **D**, Week 12: CST < 325 μm, extend to Q8W; week 20: CST within ±10% of reference CST (with no associated ≥10-letter BCVA decrease from reference BCVA), extend to Q12W; week 32: CST within ±10% of reference CST (with no associated ≥10-letter BCVA decrease from reference BCVA), extend to Q16W; week 48: CST increased by more than 10% (with an associated ≥10-letter BCVA decrease from reference), reduce to Q8W. ∗The first CST value that is < 325 μm (defined as the central 1-mm thickness from the internal limiting membrane to Bruch’s membrane), starting at week 12. Reference CST is adjusted if CST decreases by >10% from the previous reference CST for 2 consecutive study drug dosing visits and the values obtained are within 30 μm. The CST value obtained at the latter visit serves as the new reference CST. ^†^The mean of the 3 best BCVA scores obtained at any previous active dosing visit. ETDRS = Early Treatment Diabetic Retinopathy Study.

The algorithm recognized 3 patterns, as illustrated by the treatment decision tree in Figure 2 and outlined in Table 2, using anatomic and functional measures of retinal stability. First, if CST was stable, the PTI algorithm extended the treatment interval by 4 weeks (to a maximum of every 16 weeks), unless an accompanying decline in BCVA of 10 letters or more occurred. Central subfield thickness stability was defined as being within ±10% of the reference CST. Second, with improved CST, the PTI algorithm maintained the treatment interval. This is because the patient likely continued to benefit from the existing treatment interval and a premature extension in the treatment interval could hold back any potential future gains in vision outcomes. Central subfield thickness improvement was defined as a decrease of > 10% compared with the reference CST. Third, if CST worsened, the PTI algorithm maintained or reduced the treatment interval by 4 or 8 weeks, depending on the severity of the CST worsening and change in BCVA. For example, if a patient's CST worsened by more than 20%, the treatment interval was decreased by 4 weeks if BCVA declined by fewer than 10 letters or if BCVA increased, but the treatment interval was reduced by 8 weeks if the decline in BCVA was 10 letters or more. With moderate worsening of CST (increase from reference value >10% but ≤20%), the treatment interval was reduced by 4 weeks if an associated BCVA decrease of 5 letters or more (but < 10 letters) occurred and by 8 weeks if an associated 10-letter or more BCVA decline occurred. Figure 2 illustrates the treatment decision process (see also Table 2). Some scenario examples are illustrated in Figure 3.

### Study Outcomes and Rationale

The primary objective of the 2 studies was to evaluate the efficacy of intravitreal injections of faricimab 6.0 mg on BCVA outcomes, as measured by change from baseline in BCVA at 1 year, determined as the average change in BCVA from baseline at weeks 48, 52, and 56. Considerable intersession variability in BCVA has been reported in the literature for patients with diabetic retinopathy (DR) and age-related macular degeneration, possibly because of both measurement and disease-related factors.^18^, ^25^, ^19^, ^20^, ^21^, ^22^, ^23^ Averaging BCVA change over 3 time points reduced the impact of variability between visits, thus providing a more robust measure of the true treatment effect on BCVA than measurement at a single time point. Additionally, given the design of the YOSEMITE and RHINE trials, and therefore the variation in treatment schedules, averaging also minimized the potential impact of time since last active treatment on outcomes, and therefore allowed a fairer comparison across treatment arms.

A key secondary objective was to evaluate the efficacy of faricimab on DR severity outcomes, evaluated as the proportion of patients with an at least a 2-step Diabetic Retinopathy Severity Scale improvement from baseline on the ETDRS Diabetic Retinopathy Severity Scale at week 52. Another important secondary outcome was the durability of faricimab, which was assessed by evaluating the proportion of patients receiving treatment at different intervals (e.g., every 12 weeks or every 16 weeks) in the PTI arm at 1 and 2 years and over time. Moreover, given the relevance of anatomic changes to treatment decisions in a clinical setting, as opposed to clinical trials with fixed-interval dosing, other important secondary end points were the change in CST from baseline at 1 year and over time and proportions of patients with absence of DME, intraretinal fluid, subretinal fluid, or both intraretinal and subretinal fluid over time. Other secondary objectives included evaluation of BCVA change from baseline over time, additional categorical vision end points (proportion of patients gaining and proportion of patients avoiding a loss of ≥15 letters, ≥10 letters, ≥5 letters, or ≥0 letters in BCVA from baseline over time; proportion of patients gaining ≥15 letters or achieving BCVA of ≥84 letters over time; proportion of patients with BCVA Snellen equivalent of 20/40 or better over time; and proportion of patients with BCVA Snellen equivalent of 20/200 or worse over time), DR-related outcomes (proportion of patients with ≥2-step or ≥3-step DR severity improvement on the ETDRS Diabetic Retinopathy Severity Scale over time, and the proportion of patients who demonstrate new proliferative DR over time), and 25-item National Eye Institute Visual Function Questionnaire composite score outcome at 1 year. Exploratory efficacy objectives focused on further evaluating the efficacy of faricimab on anatomic measures using SD OCT, FFA, OCT angiography, or a combination thereof and on further evaluation of patient-reported outcomes using the 25-item National Eye Institute Visual Function Questionnaire. The key pharmacokinetic objective was to characterize the systemic pharmacokinetics of faricimab. Immunogenicity analyses included antidrug antibody status at baseline and time points after baseline and the relationship with efficacy, safety, or pharmacokinetic end points. Exploratory pharmacokinetic, pharmacodynamic, and biomarker objectives were used to identify biomarkers predictive of response to faricimab or disease progression and are described further in a separate section, below.

### Safety Assessments

The safety objective of the 2 studies was to evaluate the ocular and systemic safety and tolerability of faricimab. Detailed ocular examinations, including indirect ophthalmoscopy and slit-lamp examination, were performed throughout the study to year 1 and were continued through year 2. An independent data monitoring committee proactively monitored safety and study conduct throughout the primary end point at year 1 and thereafter continued receiving regular updates and maintained access to the data through the end of the studies.

The following safety summaries for the safety-evaluable population were planned for years 1 and 2, and other time points as applicable: summaries of adverse events (AEs), AEs leading to study discontinuations, treatment-emergent and serious AEs, and deaths and descriptive summaries of ocular and laboratory test findings and vital sign abnormalities. All AEs, including serious AEs and AEs of special interest, were required to be recorded by the study site on the AE electronic case report form and to be reported to the sponsor. Intraocular inflammation and infectious endophthalmitis were AEs of special interest, based on experience with intravitreal anti-VEGF injections, and individual occurrences of these events were evaluated and documented by the study sites.

### Pharmacodynamic and Biomarker Assessments and Rationale

Blood plasma samples for pharmacodynamic evaluation were collected at day 1 and selected time points throughout the study, including the final visit or any early termination visit. Optional aqueous humor and vitreous samples (when vitrectomy was medically necessary) were collected from consenting patients at baseline and at selected time points, including any early termination visit, throughout the study for analysis of drug concentrations, antibodies to faricimab, and biomarkers. To increase our understanding of the ocular pharmacokinetics and pharmacodynamics of faricimab and their relationship to the PTI, aqueous humor samples were measured at different time points for consenting patients in centers where optional sampling was approved. Data from the analyses may be used to develop better predictive models for determining optimal dosing intervals.

Free VEGF and free angiopoietin-2 were measured in the systemic circulation as part of the pharmacodynamic assessments at different time points for all patients and at 2 separate time points for patients who consented to additional sampling, to evaluate the drug exposure–effect relationship. Biomarker assessments included exploratory analyses of biochemical entities (e.g., cytokines, interleukin-6, interleukin-1b, and monocyte chemoattractant protein-1) to investigate the role of biochemical and biological processes such as angiogenesis, inflammation, and oxidative stress in the pathogenesis of DR and in the response to faricimab treatment. The potential association of these biomarkers with disease progression may be explored to help elucidate the role of angiopoietin-2 in the pathophysiologic features of DME and DR and the potential benefits of faricimab for patient outcomes.

### Statistical Approaches

The study aimed to enroll a total sample size of approximately 300 patients per arm. This provided more than 90% power to show noninferiority of faricimab versus aflibercept (pairwise comparisons between the active comparator and each of the faricimab arms) in the intention-to-treat (ITT) population, using a noninferiority margin of 4 letters of BCVA (based on discussions with health regulatory bodies) and under the following assumptions: standard deviation of 11 letters for the change from baseline in BCVA at the 1-year end point, 2-sample *t* test, 1.25% 1-sided type I error rate, and a 10% dropout rate.

Changes in BCVA from baseline (primary outcome) were compared using a model-based approach, with a mixed model for repeated measures that included change from baseline at weeks 4 through 56 as the response variable and the categorical covariates of treatment group, visit, visit-by-treatment group interaction, and baseline BCVA (continuous), as well as randomization stratification factors as fixed effects. Missing data were imputed implicitly by the mixed model for repeated measures. Comparisons between each faricimab arm and the aflibercept every 8 weeks arm were made using the estimated difference across treatment arms in the average over weeks 48, 52, and 56.

The 1-year outcome was the average of the week 48, 52, and 56 visits. Statistical analysis used a graph-based testing procedure to control for the overall type I error rate.^24^ For each faricimab arm versus aflibercept, 3 hypotheses were tested in hierarchical order at an overall significance level of α = 0.0496: noninferiority of faricimab 6.0 mg every 8 weeks or PTI compared with aflibercept 2.0 mg every 8 weeks in the ITT population (noninferiority margin of 4 letters), superiority of faricimab 6.0 mg compared with aflibercept 2.0 mg every 8 weeks in the treatment-naïve population, and superiority of faricimab 6.0 mg compared with aflibercept 2.0 mg every 8 weeks in the ITT population (Supplementary Fig 1).

Durability outcomes in the PTI arm were evaluated by descriptively showing the proportion of individuals assigned to different treatment intervals, from every 4 weeks to every 16 weeks, at the week 52 study visit. If a patient received a study treatment at the week 52 visit, the treatment interval used for analysis was that derived by IxRS using the BCVA and CST data from that study visit. Thus, if a patient was receiving treatment at the every-8-week interval coming into the week 52 study visit and showed BCVA and CST values that led to IxRS extending the treatment interval to every 12 weeks, the every-12-week interval was used to describe the patient’s treatment interval at the primary end point. Conversely, if a patient was receiving treatment at the every-16-week interval coming into the week 52 study visit, but showed BCVA and CST values that led to IxRS reducing the treatment interval to every 12 weeks at week 52, the every-12-week interval was used to denote the patient’s treatment interval at the primary end point.

## Study Status

The YOSEMITE trial commenced recruitment in September 2018, and primary end point completion occurred in October 2020. The RHINE trial commenced recruitment in October 2018, and completion of the primary end point was in October 2020. The global periods of the core studies were completed in August and September 2021, respectively.

## Discussion

The phase 3 YOSEMITE and RHINE trials for DME were primarily designed to show noninferiority of faricimab compared with aflibercept in the ITT population, which included both anti-VEGF treatment-naïve and previously treated patients. In addition to faricimab efficacy, safety, and pharmacokinetics, the objectives included assessing extended durability of faricimab, a novel anti–angiopoietin-2 and anti-VEGF bispecific antibody constructed using CrossMAb technology (F. Hoffmann-La Roche Ltd., Basel, Switzerland) designed specifically for intraocular use,^25^ compared with a fixed-interval aflibercept regimen dosed per the prescribing information.^13^, ^14^, ^15^ To address heterogeneity of treatment response in DME, the studies incorporated a PTI arm that used a protocol-specified regimen, with 4-week treatment interval extensions up to every 16 weeks, designed based on the T&E concept.

In preclinical models, dual inhibition of angiopoietin-2 and VEGF with faricimab was shown to synergistically promote vascular stability and reduce vascular leakage compared with intravitreal anti-VEGF monotherapy.^25^ Furthermore, dual angiopoietin-2 and VEGF inhibition in preclinical models led to anti-inflammatory effects, including reduced inflammatory cell infiltration of the retina compared with anti-VEGF monotherapy. The potential for extended durability with faricimab was shown in the phase 2 clinical trials that evaluated faricimab in comparison with ranibizumab.^17^^,^^18^^,^^26^

The burden of intravitreal anti-VEGF monotherapy and need for frequent clinic visits for treatment, monitoring, or both is high for patients with DME, their caregivers, and health care providers.^27^^,^^28^ Despite the efficacy shown with intravitreal anti-VEGF monotherapy in clinical trials with monthly visits, many patients with DME do not achieve and maintain similar outcomes in settings outside of clinical trials, with many not experiencing clinically meaningful improvements in vision over the long term, where monthly treatment and monitoring is not feasible.^7^^,^^8^^,^^29^ Personalized treatments have been evaluated in the past (PRN, T&E), but large-scale data from a diverse population are limited on the efficacy of an automated, standardized T&E algorithm based on BCVA and OCT data. Furthermore, T&E regimens in DME were previously evaluated only for treatment intervals up to every 12 weeks.^9^^,^^11^^,^^12^

The benefits and limitations of current T&E strategies are exemplified in 2 clinical ranibizumab trials. The RETAIN trial compared T&E with PRN ranibizumab 0.5 mg in DME.^12^ It used monthly visits, and study investigators determined treatment intervals based on functional BCVA. Patients in the T&E group achieved similar improvements in BCVA compared with those in the PRN group, but made only 8.9 scheduled visits compared with 16.6 scheduled visits for the PRN group.^12^ The TREX-DME trial compared a T&E regimen with monthly treatments, with the T&E protocol using an algorithm largely based on CST changes (evaluated and calculated at the individual site level) and using 2-week interval extensions.^11^ Patients enrolled in the T&E regimen showed similar visual and anatomic outcomes as those enrolled in the monthly regimen and a reduction of 2.4 treatments per year (10.7 injections in the T&E group compared with 13.1 injections in the monthly ranibizumab group).

The VIBIM trial (intravitreal aflibercept in DME) evaluated a T&E regimen with aflibercept. This was a small single-arm study conducted in South Korea that included a T&E phase, during which dosing intervals could be changed by 2 weeks based on changes in CST.^30^ The treatment interval was shortened by 2 weeks if CST increased by at least 10%, was extended by 2 weeks if CST was stable, or was maintained if CST was reduced by at least 10%. The results at 1 and 2 years showed that flexible dosing could avoid overtreatment.^30^^,^^31^

**Figure 4:**
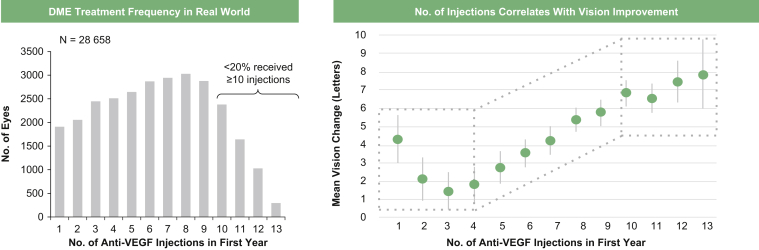
Graphs showing that low anti–vascular endothelial growth factor (VEGF) injection frequency in clinical practice versus clinical trials correlates with suboptimal outcomes. Adapted from Ciulla TA, Pollack JS, Williams DF. *Br J Ophthalmol*. 2021;105:216–221.^29^ Adapted under the terms of the CC BY-NC 4.0 license. DME = diabetic macular edema.

Together, despite the clear efficacy of T&E regimens, these studies highlight key limitations, including frequent office visits, subjective treatment decisions, the need to manually calculate changes in treatment criteria over time, and relatively short extension times of 2 weeks. The frequency of scheduled visits is particularly challenging because a large body of evidence from neovascular age-related macular degeneration and DME trials has demonstrated that the treatment visit schedules underlying clinical trials are difficult to translate into a clinical practice setting, an issue potentially compromising treatment efficacy (Fig 4).^7^^,^^8^^,^^29^^,^^32^^,^^33^ We have tried to address many of these challenges in the setting of a registrational trial with YOSEMITE and RHINE, which were designed to objectively evaluate the benefits of an automated, protocol-determined, prespecified regimen, adjustable up to every 16 weeks, based on the concept of T&E. Two faricimab regimens were tested: the conventional fixed every-8-week regimen (as in the comparator arm) and individualized IxRS-guided PTI dosing that allows for incremental changes by 4 weeks up to a maximum of every 16 weeks, with reductions by 4 and 8 weeks if needed. The PTI design was enabled by time to disease reactivation and pharmacokinetic data from the phase 2 BOULEVARD trial, which suggested that faricimab patients experienced greater durability of effect, with greater average times to disease reactivation in the off-treatment period, compared with anti-VEGF monotherapy. In addition to the objectivity and standardization of dosing in the PTI arm, another key improvement relative to previous T&E regimens was that instead of 2-week increases, PTI extensions were by 4 weeks, and instead of a maximum every-12-week interval, the PTI allowed a maximum interval extension of every 16 weeks. Indeed, a T&E regimen that is largely based on 4-week extensions or reductions could help to reduce the frequency of scheduled visits and could improve outcomes in clinical practice.

The TREX-DME algorithm^11^ and the PTI treatment algorithm used in the YOSEMITE and RHINE trials have some similarities and some notable differences. In both algorithms, treatment intervals could be extended from week 12 if the CST was < 325 μm (note, the terminology used in the TREX-DME trial was “central retinal thickness,” but the definition was consistent with how we have defined CST: the average retinal thickness of the central 1 mm around the fovea). Treatment intervals could be extended or reduced by 2 weeks in the TREX-DME trial during the T&E phase after the retinal thickness criteria were met, but by 4 weeks in the PTI algorithm in the YOSEMITE and RHINE trials. In the TREX-DME trial, the retinal thickness measurement at the time of treatment extension was used as the baseline for determining treatment interval modifications from that point onward, and a new baseline retinal thickness was established if the retinal thickness had improved by more than 20% from baseline for 3 consecutive visits and variability was < 50 μm among those 3 visits. The adjustments to reference retinal thickness were more sensitive to changes in the PTI algorithm; the reference CST was adjusted if CST decreased by more than 10% from the previous reference CST for 2 consecutive study drug dosing visits and the values obtained were within 30 μm of each other. Loss of BCVA letters above set criteria resulted in reduction of the treatment interval in both the TREX-DME trial and the PTI algorithms. In the TREX-DME trial, the treatment interval was automatically reduced to 4 weeks if 15 letters were lost because of DME.^11^ The PTI algorithm specified reductions to treatment interval by 4 or 8 weeks, depending on CST change and the severity of BCVA worsening.

The value of 2 identically designed, double-masked, randomized studies is that they are able to demonstrate consistency and reproducibility in outcomes. To our knowledge, YOSEMITE and RHINE are the first phase 3 registration trials in DME to incorporate an automated protocol-prespecified PTI dosing regimen that is based on the widely used T&E concept^16^ in a double-masked manner. The trial design aligns with clinical practice in that treatment decisions during the PTI phase were made only at the dosing visits and not at the sham visits and were based on both functional and anatomic assessments. The PTI was automated, standardized, and objective and was used across a large global DME population to test the efficacy and durability benefits of the PTI approach in a diverse group of patients. The value of this approach was to limit any subjective decision-making around the treatment interval. Although the use of CRCs with masked graders constituted a strength of the studies in the context of providing unbiased and objective clinical evaluation, the value of such an automated approach in clinical practice requires further investigation.

The YOSEMITE and RHINE trials thus should provide further insights on the efficacy and safety of faricimab for the treatment of DME, as well as on the potential benefits of an algorithm-based PTI regimen allowing for up to every-16-week dosing with faricimab. The 2-year outcomes will allow a deeper assessment of the potential for faricimab, through dual angiopoietin-2 and VEGF-A inhibition, to improve retinal stability and to deliver sustained efficacy in the long term. Results from the YOSEMITE and RHINE trials, particularly those at 2 years, will also show whether the PTI approach to dosing has the potential to reduce the burden of frequent visits and injections while maintaining vision outcomes through individual optimization of treatment intervals. The prespecified and automatically assigned interval adjustments (with potential for dosing intervals of up to every 16 weeks) based on CST and BCVA at dosing visits, and regardless of the CST and BCVA measurements at sham visits, are key features of the study design that will support this evaluation.

Faricimab is also under evaluation for the treatment of neovascular age-related macular degeneration in 2 ongoing phase 3 trials, TENAYA and LUCERNE, and for the treatment of macular edema resulting from retinal vein occlusion in 2 ongoing phase 3 trials, BALATON and COMINO. In the TENAYA (ClinicalTrials.gov identifier, NCT03823287) and LUCERNE (ClinicalTrials.gov identifier, NCT03823300) trials, the efficacy, safety, and durability of faricimab in patients with neovascular age-related macular degeneration is being investigated using an individualized treatment interval of up to every 16 weeks, based on disease activity assessment, compared with aflibercept every 8 weeks. In the BALATON (ClinicalTrials.gov identifier, NCT04740905) and COMINO (ClinicalTrials.gov identifier, NCT04740931) trials, the efficacy, safety, and durability of faricimab in patients with macular edema resulting from branch and central retinal vein occlusion, respectively, is being investigated during the initial 6-month every-4-week dosing period compared with aflibercept dosed every 4 weeks, followed by faricimab PTI dosing, with adjustable intervals up to every 16 weeks, based on CST and BCVA at dosing visits.
