# Supplementary material for: YOSEMITE and RHINE: Phase 3 Randomized Clinical Trials of Faricimab for Diabetic Macular Edema: Study Design and Rationale
Source: Ophthalmol Sci. 2021 Dec 30;2(1):100111. doi: 10.1016/j.xops.2021.100111 (PMC9559760; doi:10.1016/j.xops.2021.100111)

Figure S1. Graph-based testing procedure for the primary end point for faricimab 6.0 mg administered either every 8 weeks (Q8W) or per personalized treatment interval (PTI) versus aflibercept 2.0 mg Q8W. α = 0.0496.

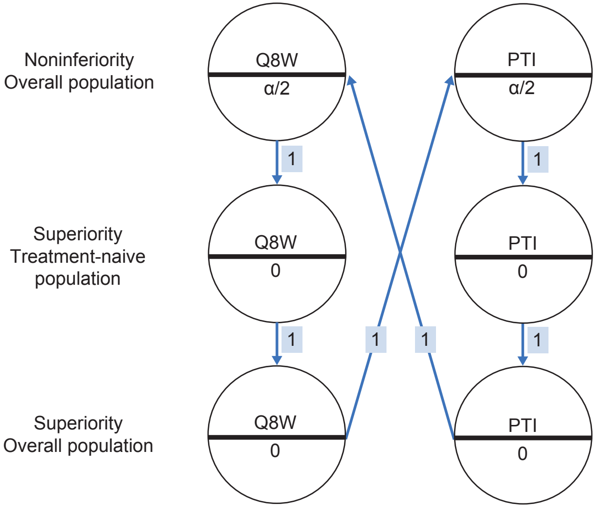

Supplement: Figure S1 [file mmc1.pdf]
