# Supplementary material for: YOSEMITE and RHINE: Phase 3 Randomized Clinical Trials of Faricimab for Diabetic Macular Edema: Study Design and Rationale
Source: Ophthalmol Sci. 2021 Dec 30;2(1):100111. doi: 10.1016/j.xops.2021.100111 (PMC9559760; doi:10.1016/j.xops.2021.100111)
Supplement: Table S1 [file mmc2.pdf]

Supplementary Information

eTable S1. Full List of institutional review boards (IRB)/ethics committees (EC) for YOSEMITE and RHINE trials

Investigator	IRB/EC Name and Address	Country
Chow, David<br>Lalonde, Laurent<br>Sheidow, Thomas	Advarra Inc., 300-372 Hollandview Trail, L4G 0A5, AURORA, Ontario, CANADA	Canada
Aaberg Jr., Thomas<br>Abbey, Ashkan<br>Abraham, Prema<br>Adams, Serrhel<br>Alam, Suhail<br>Alfaro, Virgil<br>Almony, Arghavan<br>Amini, Payam<br>Antoszyk, Andrew<br>Awh, Carl C.<br>Baker, Carl<br>Barakat, Mark<br>Bertolucci, George<br>Bochow, Thomas<br>Boyer, David<br>Brown, David M.<br>Brown, Jamin<br>Burgess, Stuart<br>Busquets, Miguel<br>Carlson, John<br>Castellarin, Alessandro<br>Chan, Clement<br>Chang, Emmanuel<br>Chang, Jonathan<br>Charles, Steve<br>Chaudhry, Nauman<br>Chen, Judy<br>Connolly, Brian<br>Danzig, Carl<br>Dessouki, Amr<br>Do, Brian<br>Eichenbaum, David<br>Engstrom, Robert<br>Falk, Naomi<br>Feiner, Leonard<br>Ferrone, Philip	Advarra, 6940 Columbia Gateway Drive, COLUMBIA, MD, 21046, UNITED STATES	United States of America

Fine, Howard
Fox, Gregory M.
Foxman, Scott
Ghorayeb, Ghassan
Gonzalez, Victor
Gupta, Sunil
Heier, Jeffrey
Hershberger, Vrinda
Higgins, Patrick
Holekamp, Nancy
Hsu, Jason
Hu, Allen
Javey, Golnaz
Javid, Cameron
Kapoor, Kapil
Khanani, Arshad
Kuriyan, Ajay
Kwong, Henry
Kwun, Robert
Laird, Philip
Lee, Seong
Liu, Mimi
London, Nikolas
Makkouk, Fuad
Malik, Khurram
Marcus, Dennis
Margherio, Alan
Maturi, Raj
McCabe, Frank
Moore, Jeffrey
Newell, Charles
Nielsen, Jared
Oh, Kean
Osher, James
Parke, D. Wilkin
Patel, Sugat
Patel, Sunil
Pearlman, Joel
Perkins, Stephen
Qureshi, Jawad
Rathod, Rajiv
Rofagha, Soraya
Rosberger, Daniel
Schadlu, Ramin
Shah, Rohan
Shah, Sandeep
Sheth, Veeral
Singer, Michael

Singerman, Lawrence		
Spinak, David-J		
Steinle, Nathan		
Stern, Jeffrey		
Stoller, Glenn		
Stoltz, Robert		
Stone, Cameron		
Suan, Eric		
Sun, Jennifer		
Suner, Ivan		
Tlucek, Paul		
Torti, Robert		
Uchiyama, Eduardo		
Weber, Pamela		
Wee, Raymond		
Weishaar, Paul		
Wells, John A.		
Williams, Thomas Reginald		
Wolfe, Jeremy		
Wykoff, Charles C.		
Zheutlin, Jeffrey		
Adrean, Sean		
Bergstrom, Chris		
Goldberg, Roger		
Khurana, Rahul		
Liu, Judy		
Pieramici, Dante		
Raskauskas, Paul		
Rosenblatt, Brett		
Shah, Ankur		
Tabassian, Ali		
Taylor, Stanford		
Thompson, John		
Wong, Robert		
Fukutomi, Akira	Aichi Medical University Hospital Institutional Review Board, 1-1 Yazakokarimata Nagakute-shi, 480-1195 Aichi, JAPAN	Japan
Karabas, Levent<br>Ozcalışkan, Sehnaz<br>Sermet, Figen<br>Yilmaz, Gursel	Ankara Universtiesi Tıp Fakultesi Klinik Arastırmalar Etik Kuurlu	Turkey
Shimouchi, Akito	Asahikawa Medical University Hospital Institutional Review Board, 1-1-1 Midorigaokahigashi2jo,Asahikawa-shi, 078-8510, Hokkaido, JAPAN	Japan

Yoon, Young Hee	Asan Medical Center Ethics Committee, 88, Olympic-ro 43-gil, Songpa-gu, 05505, Seoul, KOREA, REPUBLIC OF	Republic of Korea
Asaria, Riaz<br>Burton, Ben<br>Ghanchi, Faruque<br>Jackson, Tim<br>Jafree, Afsar<br>Lotery, Andrew<br>McKibbin, Martin<br>Menon, Geeta<br>Pearce, Ian<br>Ross, Adam<br>Stone, Amy<br>Talks, James<br>Varma, Deepali<br>Esposti, Simona<br>Harris, Martin<br>Mohamed, Quresh	Brighton and Sussex University Hospitals NHS Trust; Research and Development Department	United Kingdom
Cheong-Leen, Richard<br>Peto, Tunde	Brighton and Sussex University Hospitals NHS Trust; Research and Development Department, Royal Sussex County Hospital, Eastern Road, Brighton, BN2 5BE, UNITED KINGDOM	United Kingdom<br>United Kingdom
Mastropasqua, Leonardo	CE Uni G. D`Annunzio Asl 2 Lanciano-Vasto-Chieti, Via dei Vestini 31, 66100, Chieti, Abruzzo, ITALY	Italy
Lozano Rechy, David<br>Ramirez Estudillo, Juan	CEI de Clinica Bajio CLINBA; Comite de Etica en Investigacion, Calle Doctor Tomás Zavala No. 47, Col. Burócrata, Guanajuato, Gto., CP 36256, 36256, MARFIL, GUANAJUATO, MEXICO	Mexico
Garcia, Renata	CEI Instituto Mexicano de Oftalmologia; Comite de Etica en Investigacion, Circuito Exterior Estadio Corregidora s/n Col. Colinas del Cimatario, 76090, QUERÉTARO, QUERETARO, MEXICO	Mexico
Figueira, Joao<br>Gomes, Nuno<br>Silva, Rufino<br>Vaz-Pereira, Sara	CEIC - Comissão de Ética para Investigação Clínica	Portugal
Aliseda, Daniel<br>Desco, Carmen<br>Escobar, Joan Josep<br>Fernandez Vega, Alvaro<br>Figueroa, Marta<br>Montero, Javier<br>Sararols, Laura<br>Arias, Luis<br>Gallego-PInazo, Roberto	CEIC Hospital Clinico San Carlos, Farmacologia/1 planta Norte. Puerta G., Professor Martin Lagos s/n, 28040, Madrid, MADRID, SPAIN	Spain

Abengoechea, Santiago<br>Adan Civera, Alfredo<br>Amat, Pedro<br>Cabrera, Francisco<br>Cava, Carlos<br>Garcia-Layana, Alfredo<br>Gomez Ulla, Francisco<br>Ruiz Moreno, Jose Maria<br>Vela, Jose Ignacio	CEIC HU Puerta de Hierro de majadahonda, Planta 1ª - Pasillo Unidades Administrativas (peines 6-7), 28222, Majadahonda Madrid, MADRID, SPAIN	Spain
Taleb, Alexandre	CEP Hospital de Urgências de Goiânia; CEP Hospital de Urgências de Goiânia, Avenida 31 de Março esq. c/5 radial s/n° Setor Pedro Ludovico, 74820-200, Goiânia, GO, BRAZIL	Brazil
Cagini, Carlo	CER UMBRIA-COMITATO ETICO REGIONALE UMBRIA, Via M. Angeloni 61, 06124, Perugia, Umbria, ITALY	Italy
Pertile, Grazia	CESC DELLE PROVINCE DI VERONA E ROVIGO, Piazzale A. Stefani, 1, 37126, Verona, Veneto, ITALY	Italy
Lai, Chi-Chun	Chang Gung Med Found, Institutional Review Board	Taiwan
Sharma, Sumit	Cleveland Clinic Florida; Cleveland Clinic Instituaitonal Review Board, 9500 Euclid Avenue OS-1, Cleveland, OH, 44195, UNITED STATES	United States of America
Lavinsky, Daniel	Comissão Científica e Comissão de Pesquisa e Ética em Saúde HCPA; Grupo de Pesquisa e Pós-Graduação, Rua Ramiro Barcelos, 2350 - 2º andar, 90035-003, Porto Alegre, RS, BRAZIL	Brazil
Zacharias, Leandro	Comissão de Ética para Análise de Projetos de Pesquisa - CAPPesq, Avenida Doutor Ovídio Pires de Campos, 225, 5 andar, Prédio da Administração, 05403-010, São Paulo, SP, BRAZIL	Brazil
Giansanti, Fabrizio	COMITATO ETICO AREA VASTA CENTRO, Largo Brambilla, 3, Pad. 3 Nuovo Ingresso Careggi (NIC), 50134, Firenze, Toscana, ITALY	Italy
Parravano, Maria Cristina	Comitato Etico Centrale Sezione IFO - Bietti Istituti Fisioterapici Ospitalieri, Via Elio Chianesi, 53, edificio centrale - 1° piano, 00128, Roma, Lazio, ITALY	Italy
Bandello, Francesco	Comitato Etico Irccs Ospedale San Raffaele, VIA OLGETTINA 60, 20132, MILANO, Lombardia, ITALY	Italy
Viola, Francesco	Comitato Etico Milano Area 2, Via Francesco Sforza, 28, 20122, MILANO, Lombardia, ITALY	Italy
Lanzetta, Paolo	Comitato Etico Regionale Unico CERU, P.le S. Maria della Misericordia, 15, Pad 10 - 3° Piano, 33100, Udine, Friuli-Venezia Giulia, ITALY	Italy

Nicolo, Massimo	Comitato Etico Regione Liguria (Sezione 2), LARGO ROSANNA BENZI 10, 16132, GENOVA, Liguria, ITALY	Italy
Nardi, Marco	Comitato Etico Regione Toscana - Area Vasta Nord Ovest, Via Roma 67, c/o Presidio Ospedaliero, 56126, Pisa, Toscana, ITALY	Italy
Penha, Fernando	Comitê de É tica na Pesquisa em Seres Humanos FURB, Rua Antonio da Veiga, 140 - Câmpus 1 - Sala A-218, 89030-903, Blumenau, SC, BRAZIL	Brazil
Neto, Julio<br>Salomão, Gustavo	Comitê de Ética em Pesquisa da Faculdade de Medicina do ABC, Avenida Lauro Gome, 2000 - Vila Sacadora Cabral, 09060-870, Santo Andre, SP, BRAZIL	Brazil
Avila, Marcos	Comitê de Ética em Pesquisa do Hospital das Clínicas da Universidade Federal de Goiás, 1ª Avenida, s/n - Setor Leste Universitário - 1° andar, 74650-050, Goiânia, GO, BRAZIL	Brazil
Bordon, Arnaldo	Comite de Etica em Pesquisa do Hospital Oftalmologico de Sorocaba, Rua Nabeck Shiroma, 210, 18031-060, Sorocaba, SP, BRAZIL	Brazil
Alezzandrini, Arturo<br>Zambrano, Alberto<br>Zeolite, Carlos<br>Schlottmann, Patricio	Comite de Etica en Investigacion Clinica (CEIC), Larrea 1381 3° A, C1117ABK, Ciudad Autonoma de Buenos Aires, ARGENTINA	Argentina
Morales Canton, Virgilio	Comité de ëtica en Investigación de la Asociación para Evitar la Ceguera en México I.A.P.; CEI, Vicente García Torres No. 46, 04030, CIUDAD DE MÉXICO, MEXICO CITY (FEDERAL DISTRICT), MEXICO	Mexico
Furno Sola, Federico<br>Bafalluy, Joaquin	Comite de Etica Independiente Consultorios Integrados (CEICI), Italia 424, 2000, Rosario, Santa Fe, ARGENTINA	Argentina
Olivier, Sebastien	Comité d'éthique de la recherche HMR, 5414 boul de l'Assomption, H1T 2M4, Montreal, Quebec, CANADA	Canada
Barraza, Karen<br>Fernandez, Carlos<br>Guzman, Miguel<br>Lujan, Silvio	Comite Institucional de Bioetica Via Libre, Jr. Paraguay 478, LIMA 01, Lima, PERU	Peru
Buffet, Sylvia<br>Razavi, Hessam<br>Souied, Eric<br>Cornut, Pierre Loic<br>Korobelnik, Jean Francois<br>Lebreton, Olivier<br>Tadayoni, Ramin	CPP Ile de France V, Hôpital Saint-Antoine, 184 Rue du Faubourg Saint-Antoine, 75012, Paris, FRANCE	France
Vajas, Attila	DE RKEB/IKEB, Nagyerdei krt. 98, 4032, DebrecenDebrecen, HUNGARY	Hungary

Larsen, Michael<br>Laugesen, Caroline<br>Vorum, Henrik	De Videnskabsetiske Komitéer for Region Hovedstade	Denmark
Kulikov, Alexey	EC of FSB Military educational institution of HPE "Military Medical Academy n.a. S. M, Academic Lebedeva str., 6, 194044, Saint-Petersburg, RUSSIAN FEDERATION	Russia
Kerenyi, Agnes	Egeszsegugyi Tudomanyos Tanacs, Arany Janos u. 6-8, 1051, Budapest, HUNGARY	Hungary
Lorenz, Katrin	EK Rheinland-Pfalz LÄK, Deutschhausplatz 3, 55116, Mainz, GERMANY	Germany
Seitz, Berthold	EK Saarland LÄK, Faktoreistraße 4, 66111, Saarbruecken, GERMANY	Germany
Eter, Nicole<br>Spital, Georg	EK Westfalen-Lippe ÄK, Gartenstr. 210 - 214, 48147, Münster, GERMANY	Germany
Kampik, Daniel	EK Würzburg, Versbacher Str. 9, 97078, Würzburg, GERMANY	Germany
Schuart, Claudia	EK-Uni Magdeburg, Leipziger Str. 44, 39120, Magdeburg, GERMANY	Germany
Daskalov, Vesselin<br>Misheva, Aneta<br>Petkova, Iva<br>Tosheva Guneva, Daniela<br>Vassileva, Petja	Ethics Committee for Multicenter Trials, 8 Damyan Gruev Str., 1303, Sofia, BULGARIA	Bulgaria
Hurcikova, Maria<br>Kacerík, Marek<br>Lipkova, Blandina	Ethics Committee of the Hospital Žilina, ul. V. Spanyola 43, 012 07, Žilina, SLOVAKIA	Slovakia
Sandner, Dirk	Ethik-Kommission am Universitätsklinikum Carl-Gustav-Carus Technische Universität Dresden, Fetscherstrasse 74, 01307, Dresden, GERMANY	Germany
Bolz, Matthias	Ethikkommission d. Landes Oberösterreich, Wagner-Jauregg-Weg 15, 4020, Linz, AUSTRIA	Austria
Agostini, Hansjurgen	Ethik-Kommission der Albert-Ludwigs-Universität, Engelberger Straße 21, 79106, Freiburg, GERMANY	Germany
Lohmann, Chris P.	Ethikkommission der Fakultaet fuer Medizin der Tec, Ismaninger Str. 22, 81675, Muenchen, GERMANY	Germany
Weger, Martin	Ethikkommission der Med. Univ. Graz, Auenbruggerplatz 2, Eingangsgebäude, 3. OG, 8036, Graz, AUSTRIA	Austria
Priglinger, Siegfried	Ethik-Kommission der Medizinischen Fakultaet der L, Pettenkoferstr. 8a, 80336, Muenchen, GERMANY	Germany

Framme, Carsten	Ethik-Kommission der Medizinischen Hochschule Hannover, Carl-Neuberg-Str. 1, 30625, Hannover, GERMANY	Germany
Findl, Oliver	Ethikkommission der Stadt Wien gemäß KAG, AMG und MPG, TownTown, Thomas-Klestil-Platz 8, 1030, Wien, AUSTRIA	Austria
Pollreisz, Andreas	Ethikkommission der Universität Wien /AKH, Borschkegasse 8b/E 06, 1090, Wien, AUSTRIA	Austria
Feltgen, Nicolas	Ethikkommission der Universitätsmedizin Göttingen, Von-Siebold-Str. 3, 37075, Göttingen, GERMANY	Germany
Hatz, Katja	Ethikkommission Nordwest- und Zentralschweiz (EKNZ), Hebelstrasse 53, 4056, Basel, SWITZERLAND	Switzerland
Ernest, Jan	Eticka komise Axon Clinical s.r.o, Matouse Sevce 26, 140 00, Praha, CZECH REPUBLIC	Czech Republic
Nemcansky, Jan	Eticka komise Fakultni nemocnice Ostrava, 17 Listopadu 1790, 70852, Ostrava, CZECH REPUBLIC	Czech Republic
Ernest, Jan<br>Farkas, Andrej<br>Nemcansky, Jan<br>Veith, Miroslav<br>Dusova, Jaroslava	Etická komise FNKV, Šrobárova 50, 100 34, Prague 10, CZECH REPUBLIC	Czech Republic
Hurcikova, Maria	Eticka komisia Nemocnice s poliklinikou Trebisov,a.s., SNP 1079/76, 075 01, Trebišov, SLOVAKIA	Slovakia
Kacerík, Marek	Eticka Komisia pri FN Trencin, Legionarska 28, 911 71, Trencin, SLOVAKIA	Slovakia
Dusova, Jaroslava	Fakultni nemocnice Hradec Kralove; Eticka komise, Sokolska 581, 500 05, Hradec Kralove, CZECH REPUBLIC	Czech Republic
Ricci, Federico	Fondazione PTV Policlinico Tor Vergata; Comitato Etico Indipendente, Viale Oxford, 81, 00133, Roma, Lazio, ITALY	Italy
Budzinskaya, Maria	FSBI "Scientific Research Institute of Eye Diseases" of russia Academy of medical Sciences	Russia
Eldem, Bora<br>Mentes, Jale<br>Ozturk, Banu	Hacettepe University Ethics Committee; Ethics Committtee	Turkey
Obana, Akira	Hamamatsu Clinical Research Network Institutional Revew Board, 2-12-12 Sumiyoshi, Naka-Ku, Hamamatsu shi, 430-8558, Shizuoka, JAPAN	Japan
Hayashi, Ken	Hayashi Eye Hospital Institutional Review Board, 4-23-35 Hakata-eki mae, Hakata-ku, 812-0011, Fukuoka, JAPAN	Japan
Williams, Geoff	Health Research Ethics Board of Alberta, 1500, 10140 - 103 Avenue NW, T5J-1V3, Edmonton, Alberta, CANADA	Canada
Rosenblatt, Irit	Helsinki Committee - Rabin Beilinson M.C	Israel

Yoreh, Barak	Helsinki Committee – Rambam	Israel
Levy, Jaime	Helsinky committee Hadassah EK	Israel
Fung, Nicholas	HKU/HA HKW IRB	Hong Kong
Noda, Kousuke	Hokkaido University Hospital Institutional Review Board, Kita14-jo,Nishi5-chome,Kita-ku,Sapporo, 060-8648, Hokkaido, JAPAN	Japan
Oh, Hideyasu	Hyogo Prefectural Amagasaki General Medical Center (Hyogo AGMC) IRB, 2-17-77 Higashinaniwa-cho, amagasaki-shi, 660-8550, Hyogo, JAPAN	Japan
Pongsachareonnont, Pear	Institutional Review Board, Faculty of Medicine, 3rd floor, Ananda Mahidol Building,, 10330, Patumwan, Bangkok, THAILAND	Thailand
Takagi, Hitoshi	IRB of a group of St. Marianna Univ. School of Medicine Hospitals, 2-16-1 Sugao,Miyamae-ku, Kawasaki- shi, 216-8511, Kanagawa, JAPAN	Japan
Campochiaro, Peter	Johns Hopkins Medicine Institutional Review Board, 1620 McElderry Street, Reed Hall B-130, Baltimore, MD, 21205-1911, UNITED STATES	United States of America
Hau, Vivienne	Kaiser Permanente Southern California, 393 East Walnut Street, 2nd Floor, Pasadena, CA, 91188, UNITED STATES	United States of America
Morori-Katz, Haia	Kaplan Medical Center Ethics Committee	Israel
Sikorski, Bartosz<br>Kaluzny, Jakub	Komisja Bioetyczna przy Bydgoskiej Izbie Lekarskiej, Powstancow Warszawy 11, 85-681, Bydgoszcz, POLAND	Poland
Muzyka-Wozniak, Maria	Komisja Bioetyczna przy Dolnoslaskiej Izbie Lekarskiej, Al. Jana Matejki 6, 50-333, Wroclaw, POLAND	Poland
Borcz, Emilia<br>Kaluzny, Jakub<br>Romanczak, Dominika<br>Romanowska-Dixon, Bożena<br>Sikorski, Bartosz<br>Zatorska, Barbara<br>Raczynska, Dorota<br>Zaczek Zakrzewska, Karolina	Komisja Bioetyczna przy Okręgowej Izbie Lekarskiej w Gdańsku, Śniadeckich 33, 80-204, Gdansk, POLAND	Poland
Nester-Ostrowska, Kamila	Komisja Bioetyczna przy Okregowej Izbie Lekarskiej w Rzeszowie, ul. Jana Dekerta 2, 35-030, Rzeszów, POLAND	Poland

Gawecki, Maciej<br>Herba, Ewa<br>Michalska- Malecka, Katarzyna<br>Muzyka-Wozniak, Maria<br>Nester-Ostrowska, Kamila<br>Oleksy, Piotr<br>Wowra, Bogumil	Komisja Bioetyczna przy Slaskiej Izbie Lekarskiej w Katowicach, 49a, Grazynskiego, 40-126, Katowice, POLAND	Poland
Wylęgała, Edward	Komisja Bioetyczna przy Slaskiej Izbie Lekarskiej w Katowicach, 49a, Grazynskiego, 40-126, Katowice, POLAND	Poland
Kishino, Genichiro	Kozawa eye hospital and diabetes center IRB, 246-6, Yochizawa-cho, Mito-shi, 310-0845, Ibaraki, JAPAN	Japan
Yoshida, Shigeo	Kurume University Institutional Review Board, 67 Asahimachi, Kurume-shi, 830-0011, Fukuoka, JAPAN	Japan
Hirakata, Akito	Kyorin University Hospital Institutional Review Board, 6-20-2 Shinkawa, Mitaka, 181-8611, Tokyo, JAPAN	Japan
Murakami, Tomoaki	Kyoto University Hospital Institutional Review Board, 54 Kawaharacho, Shogoin, Sakyo-ku, 606-8507, Kyoto, JAPAN	Japan
Yu, Seung Young	Kyung Hee University Hospital; IRB, 23, Kyungheedae-ro, Dongdaemun-gu, 02447, Seoul, KOREA, REPUBLIC OF	Republic of Korea
Bratko, Galina<br>Pozdeyeva, Nadezhda	LEC of "Intersec. Research and Technology Complex "Eye Microsurgery" n.a. S.N. Fyodorov	Russia
Yurieva, Tatiana	LEC of "Intersec. Research and Technology Complex "Eye Microsurgery" n a Fyodorov Irkutsk branch	Russia
Abdulaeva, Elmira	LEC of Ltd."Kuzlyar"	Russia
Seres, András<br>Vogt, Gábor	Medical Research Council, Ethics Committee for Clinical Pharmacology, Arany J. u. 6-8., 1051, Budapest HUNGARY	Hungary
Sugimoto, Masahiko	Mie University Hospital Institutional Review Board, 2-174 Edobashi, Tsu-shi, 514-8507, Mie, JAPAN	Japan
Yasukawa, Tsutomu	Nagoya City University Hospital Institutional Review Board, 1 Kawasumi Mizuho-cho, Mizuho-ku, Nagoya-shi, 467-8602, Aichi, JAPAN	Japan
Ito, Yasuki	Nagoya university Hospital IRB, 65 tsurumai-cho, showa-ku, nagoya-shi, 466-8560, Aichi, JAPAN	Japan
Ogata, Nahoko	Nara Medical University Hospital IRB	Japan
Takayama, Kei	National Defense Medical College Hospital Institutional Review Board, 3-2 Namiki, Tokorozawa-shi, 359- 8513, Saitama, JAPAN	Japan

Dickinson, John	Nova Scotia Health Authority Research Ethics Board, QEII Health Science Centre for Clinical Res, Room 118-5790 University Avenue, B3H 1V7, Halifax, Nova Scotia, CANADA	Canada
Honda, Shigeru	Osaka City University Hospital IRB, 1-5-7, Asahimachi, Abeno-ku, Osaka-shi, 545-8586, Osaka, JAPAN	Japan
Dollin, Michael	Ottawa Ethics Board; Ottawa Health Science Network Research Ethics Board (OHSNREB), 725 Parkdale Avenue, Civic Box 675, K1Y 4E9, Ottawa, Ontario, CANADA	Canada
Varsanyi, Balazs	Pecsi Tudomanyegyetem Altalanos Orvostudomanyi Kar, Rakoczi ut 2., Regionalis Kutatas-Etikai Bizottsag, 7623, Pécs, HUNGARY	Hungary
Lai, Timothy	Research Ethics Committee (Kln Central/Kln East)	Hong Kong
Yang, Chang-Hao	Research Ethics Committee, Nat. Taiwan Univ. Hosp.	Taiwan
Chaikitmongkol, Voraporn	ResearchEthics Com. Fac Med. Chiang Mai University, 110 Intavaroros Street, Amphoe Muang, 50200, Chaing Mai, THAILAND	Thailand
Wickremasinghe, Sanjeewa	Royal Victorian Eye and Ear Hospital Human Research Ethics Committee, Locked Bag 8, 8002, EAST MELBOURNE, Victoria, AUSTRALIA	Australia
Kang, Se Woong	Samsung Medical Center Institutional Review Board, 81, Irwon-ro, Gangnam-gu, 06351, Seoul, KOREA, REPUBLIC OF	Republic of Korea
Papp, Andras	Semmelweis Univ. Reg. and Instit.Committe od S&RE, Ulloi u 93, H- 1091, Budapest, HUNGARY	Hungary
Yu, HyeongGon	Seoul National Univ. Ethics Comittee, 101, Daehak-ro, Jongno-gu, Seoul, Korea, 03080, Seoul, KOREA, REPUBLIC OF	Republic of Korea
Park, Kyu Hyung	Seoul National University Bundang Hospital IRB, 82, Gumi-Ro 173 Beon-Gil, Bundang-Gu, 463-707, Seongnam-Si, Gyeonggi-Do, KOREA, REPUBLIC OF	Republic of Korea
Sun, Xiaodong	Shanghai First People's Hospital; Ethics Committee of Shanghai First People's Hospital, No.100,Haining Road.Hongkou District, 200080, Shanghai, CHINA	China
Sawada, Osamu	Shiga University of Medical Science Hospital Institutional Review Board, Seta,Tsukinowa-cho, Otsushi, 520-2192, Shiga, JAPAN	Japan
Chee, Caroline Rajagopalan, Rajesh	SingHealth Independent Review Board; Review Board A, 168 Jalan Bukit Merah, #06-08 Tower 3, Connection One, 150168, Singapore, SINGAPORE	Singapore
Tan, Gavin	SingHealth Independent Review Board; Review Board A, 168 Jalan Bukit Merah, #06-08 Tower 3, Connection One, 150168, Singapore, SINGAPORE	Singapore
Goldstein, Michaella	Sourasky Medical Centre Ethics Committee	Israel

Wong, David	St Mike's Hospital Research Ethics Board, 30 BOND STREET, M5B 1W8, TORONTO, Ontario, CANADA	Canada
Chang, Andrew<br>Chen, Fred<br>Fraser-Bell, Samantha<br>Mitchell, Paul<br>Wong, James<br>Sandhu, Sukhpal	St Vincent Human Research Ethics Committee, 41 Victoria Parade, 3065, Fitzroy, Victoria, AUSTRALIA	Australia
Sugita, Iichiro	Sugita Eye Hospital Institutional Review Board, 5-1-30, Sakae Naka-ku Nagoya-shi, 460-0008, Aichi, JAPAN	Japan
Kawasaki, Tsutomu	Sugiura Clinic Institutional Review Board, 4-4-16-301, Hon-cho, Kawaguchi-shi, 332-0012, Saitama, JAPAN	Japan
Toth-Molnar, Edit	Szegedi Tudomanyegyetem AOK; Regionalis Human Orvosbiolagiai Kutatasetikai Bizottsag, Korányi fasor 8-10, 6720, Szeged, HUNGARY	Hungary
Bator, Gyorgy	Szombathely Regionalis Kutatasetikai Bizottsag, Markusovszky Ut 3, 9700, Szombathely, HUNGARY	Hungary
Tanabe, Teruyo	Tazuke Kofukai Foundation, Medical Research Institute, Kitano Hospital Institutional Review Board, 2-4- 20 Ohgimachi Kita-Ku, Osaka-shi, 530-8480, Osaka, JAPAN	Japan
Ruamviboonsuk, Paisan	The Ethics Committee, Rajavithi Hospital, 2 Phyathai Rd. Rajthevee, 10400, Bangkok, THAILAND	Thailand
Morugova, Tatiana	The Independent Interdisciplinary Ethics Committee on Ethical Review for Clinical Studies, 51 Leningradskiy ave., Moscow, MOSKOVSKAJA OBLAST, RUSSIAN FEDERATION	Russia
Maeno, Takatoshi	Toho University Sakura Medical Center Institutional Review Board, 564-1 Shimoshizu, Sakura-shi, 285-0841, Chiba, JAPAN	Japan
Mitamura, Yoshinori	Tokushima University Hospital Institutional Review Board, 2-50-1 Kuramoto-cho, Tokushima-shi, 770-8503, Tokushima, JAPAN	Japan
Shimura, Masahiko	Tokyo Medical University Hachioji Medical Center Institutional Review Board, 1163 Tatemachi, Hachioji-shi, 193-0998, Tokyo, JAPAN	Japan

Kitano, Shigehiko	Tokyo Women's Medical University Hospital IRB, 8-1 Kawada - Cho Shinjuku-Ku, 162-8666, Tokyo, JAPAN	Japan
Baumal, Caroline	Tufts Medical Center IRB, 800 Washington Street - Box 817, Boston, MA, 02111, UNITED STATES	United States of America
Chen, Shih-Jen	TVGH Institutional Review Board	Taiwan
Ma, Patrick	UBC Clinical Research Ethics Board, Office of Research Services and Administration, 210-828 West 10th Ave., V5Z 1L8, Vancouver, British Columbia, CANADA	Canada
Yiu, Glenn	UC Davis IRB, 2921 Stockton Blvd., Suite 1429, Sacramento, CA, 95817, UNITED STATES	United States of America
Belfort Jr., Rubens	Universidade Federal de São Paulo; UNIFESP-EPM, Rua Botucatu 572 - 1º andar, Ética em Pesquisa em Seres Humanos, 04023-062, Sao Paulo, SP, BRAZIL	Brazil
Brent, Michael	University Health Network Research Ethics Board, 700 Bay Street, 17th Floor, Suite 1700, M5G 1Z6, Toronto, Ontario, CANADA	Canada
Fortun, Jorge	University of Miami, Miller School of Medicine; Human Subject Research Office, 1400 NW 10 th Avenue, Suite M809, Miami, FL, 33136, UNITED STATES	United States of America
Ikeda, Yasuhiro	University of Miyazaki Hospital Institutional Review Board	Japan
Jacoby, Rachael	University of Utah IRB, 75 South 2000 East, #111, Salt Lake City, UT, 84112, UNITED STATES	United States of America
Kim, Brian	University of Vermont Research Protections Office, University of Vermont; 213 Waterman Building, 85 South Prospect Street, Burlington, VT, 05405, UNITED STATES	United States of America
Yates, Paul	University of Virginia Institutional Review Board for Health Sciences Research, One Morton Drive, P.O. Box 800483, Charlottesville, VA, 22908, UNITED STATES	United States of America
Sheidow, Thomas	University of Western Ontario (HSREB), 1393 Western Road, N6G 1G9, London, Ontario, CANADA	Canada
Szecsko, Timea	Uzsoki Utcai Korhaz Etikai Bizottsaga, Uzsoki street 45, 1145, Budapest, HUNGARY	Hungary
Greven, Craig	Wake Forest Univ. Eye Associates; WFU Health Sciences IRB Office, 1 Medical Center Blvd, Winston-Salem, NC, 27157-1023, UNITED STATES	United States of America
Modi, Yasha<br>Das, Arup<br>Oliver, Scott<br>Ohr, Matthew	Western Institutional Review Board, 1019 39th Avenue SE, Ste 120, Puyallup, WA, 98374, UNITED STATES	United States of America
Chiang, Allen	Wills Eye Hospital IRB, 840 Walnut St., 15th Floor, Philadelphia, PA, 19107, UNITED STATES	United States of America

Kimura, Kazuhiro	Yamaguchi University Hospital Institutional Review Board, 1-1-1 Minami Kogushi Ube-Shi, 755-8505, Yamaguchi, JAPAN	Japan
Szalczer, Lajos	Zala County Hospital Committee, Zrínyi M. u. 1, 8900, Zalaegerszeg, HUNGARY	Hungary

EC = ethics committee; IRB = institutional review board.
