# Supplementary material for: YOSEMITE and RHINE: Phase 3 Randomized Clinical Trials of Faricimab for Diabetic Macular Edema: Study Design and Rationale
Source: Ophthalmol Sci. 2021 Dec 30;2(1):100111. doi: 10.1016/j.xops.2021.100111 (PMC9559760; doi:10.1016/j.xops.2021.100111)
Supplement: Table S2 [file mmc3.pdf]

Table S2. YOSEMITE and RHINE General Eligibility Criteria

**General Inclusion criteria**
• Willingness and the ability to provide signed informed consent<br>o At US sites, patients must provide HIPAA authorization, and in other countries, as applicable according to national laws<br>• Age ≥18 years<br>• Documented diagnosis of diabetes mellitus (type 1 or 2), as defined by the ADA or per WHO criteria and:<br>o Current regular use of insulin or other injectable drugs (eg, dulaglutide and liraglutide) for the treatment of diabetes, and/or<br>o Current regular use of oral anti-hyperglycemic agents for the treatment of diabetes<br>• HbA1c of ≤10% within 2 months before day 1<br>• Ability and willingness to undertake all scheduled visits and assessments<br>• For women of childbearing potential, agreement to remain abstinent (refrain from heterosexual intercourse) or use acceptable contraceptive methods that result in a failure rate of <1% per year during the treatment period and for ≥3 months after the final dose of study treatment<br>o A woman is considered to be of childbearing potential if she is post-menarcheal, has not reached a post-menopausal state (≥12 continuous months of amenorrhea with no identified cause other than menopause), and has not undergone surgical sterilization (removal of ovaries and/or uterus). The definition of childbearing potential may be adapted for alignment with local guidelines or requirements<br>o Examples of acceptable contraceptive methods include bilateral tubal ligation, male sterilization, hormonal contraceptives that inhibit ovulation, hormone-releasing intrauterine devices, and copper intrauterine devices<br>o Contraception methods that do not result in a failure rate of <1% per year, such as male or female condom with or without spermicide, and cap, diaphragm, or sponge with spermicide are not acceptable<br>o The reliability of sexual abstinence should be evaluated in relation to the duration of the clinical trial and the preferred and usual lifestyle of the patient. If a patient is usually not sexually active but becomes active, they, with their partner, must comply with the contraceptive requirements of the study
**General Exclusion criteria**

- Currently untreated diabetes mellitus or previously untreated patients who initiated oral or injectable anti-diabetic medication or insulin <3 months before day 1
- History of allergy or hypersensitivity to aflibercept and any of its excipients, fluorescein, or any study treatment-related mandatory ingredients (eg, disinfectants, anesthetics, etc) that is not amenable to treatment
- History of a severe allergic reaction or anaphylactic reaction to a biologic agent or known hypersensitivity to any component of faricimab or to aflibercept injections, study treatment procedures, dilating drops, or any of the anesthetic and antimicrobial preparations used by a patient during the study
- Active cancer within the past 12 months, except for appropriately treated carcinoma in situ of the cervix, non-melanoma skin carcinoma, and prostate cancer with a Gleason score of ≤6 and a stable prostate-specific antigen for >12 months
- Systemic treatment for suspected or active systemic infection
    - Ongoing use of prophylactic antibiotic therapy may be acceptable but must be discussed with the Medical Monitor
- Renal failure requiring renal transplant, hemodialysis, or peritoneal dialysis, or anticipated to require hemodialysis or peritoneal dialysis at any time during the study
- History of other disease, other non-diabetic metabolic dysfunction, physical examination finding, or historical or current clinical laboratory finding giving reasonable suspicion of a condition that contraindicates the use of the faricimab or aflibercept or that might affect interpretation of the results of the study, or renders the patient at high risk for treatment complications in the opinion of the investigator
- Uncontrolled blood pressure (defined as systolic >180 mmHg and/or diastolic >100 mmHg while a patient is at rest)
    - If a patient's initial reading exceeds these values, a second reading may be obtained later the same day or on another day during the screening period. If the patient's blood pressure is controlled by anti-hypertensive medication, the patient should be taking the same medication continuously for ≥30 days before day 1
- Stroke (cerebral vascular accident) or myocardial infarction <6 months before day 1
- Pregnancy or breastfeeding, or intention to become pregnant during the study
    - Women of childbearing potential must have a negative urine pregnancy test result <28 days before initiation of study treatment. If the urine pregnancy test is positive, it must be confirmed by a serum pregnancy test
- Participation in an investigational trial that involves treatment with any drug or device (with the exception of vitamins and minerals) <3 months before day 1

- Administration of systemic pro-angiogenic treatments, such as VEGF-based therapies for peripheral or coronary ischemia (eg, limb ischemia or myocardial infarction) <3 months or 5 half-lives before day 1
- Inability to comply with study or follow-up procedures
- Requirement for continuous use of any prohibited medications and treatments indicated in the study protocol

ADA = American Diabetes Association; HbA1c = glycated hemoglobin A1c; HIPAA = Health Insurance Portability and Accountability Act; WHO = World Health Organization.
